# NMR approaches in structure-based lead discovery: Recent developments and new frontiers for targeting multi-protein complexes

**DOI:** 10.1016/j.pbiomolbio.2014.08.012

**Published:** 2014-11

**Authors:** David M. Dias, Alessio Ciulli

**Affiliations:** a Department of Chemistry, University of Cambridge, Cambridge, CB2 1EW, UK; b College of Life Sciences, Division of Biological Chemistry and Drug Discovery, University of Dundee, Dow Street, DD1 5EH, Dundee, UK

**Keywords:** Structure-based lead discovery, NMR fragment screening, Hit-to-lead optimization, Protein–protein interactions, Multi-protein complexes, Protein expression

## Abstract

Nuclear magnetic resonance (NMR) spectroscopy is a pivotal method for structure-based and fragment-based lead discovery because it is one of the most robust techniques to provide information on protein structure, dynamics and interaction at an atomic level in solution. Nowadays, in most ligand screening cascades, NMR-based methods are applied to identify and structurally validate small molecule binding. These can be high-throughput and are often used synergistically with other biophysical assays. Here, we describe current state-of-the-art in the portfolio of available NMR-based experiments that are used to aid early-stage lead discovery. We then focus on multi-protein complexes as targets and how NMR spectroscopy allows studying of interactions within the high molecular weight assemblies that make up a vast fraction of the yet untargeted proteome. Finally, we give our perspective on how currently available methods could build an improved strategy for drug discovery against such challenging targets.

## 1 Introduction

A major purpose of academic and pharmaceutical drug development research is to identify natural or synthetic molecules that interact both strongly and specifically with a given biomolecule to produce an anticipated biological response (Wells and McClendon, 2007, Zorn and Wells, 2010). The majority of current drug targets are G-protein-coupled receptors, nuclear receptors, ion channels and enzymes; all of which are targets of previously adopted pharmacology-driven approaches (Makley and Gestwicki, 2013). Our understanding of the fundamentals of molecular recognition of such interactions has evolved as a result of methodological developments and technological breakthroughs over the years. The post-genomic era transformed our approach and ability to study the proteomes of many organisms and as a result “target-first pharmacology-second” strategies were established as a dominant trend (Fry, 2006, Hopkins and Groom, 2002, Makley and Gestwicki, 2013, Núñez et al., 2012, Surade and Blundell, 2012, Wells and McClendon, 2007). Recent molecular and structural biology-based and genome-based approaches have enabled identification of a plethora of biologically relevant protein–protein interactions (PPIs), many of which are established or potential targets for small molecule chemical probes and drugs (Cummings and Hamilton, 2010, Fry, 2006, Fuller et al., 2009, Higueruelo et al., 2009, Jubb et al., 2012, Nooren and Thornton, 2003, Thangudu et al., 2012, Wells and McClendon, 2007). Multi-protein complexes are responsible for numerous important biological processes and are one of the most preponderant classes of biologically active macromolecules. Targeting of PPIs within such multi-protein complexes shows promising therapeutic value but presents a number of challenges when compared with more canonical drug targets such as orthosteric enzyme active sites (Nooren and Thornton, 2003, Smith and Gestwicki, 2012). Moreover the concept of ‘druggability’ (or ligandability) has become a rational tie for target selection, as many proteins remain to be successfully obtained, manipulated and efficiently targeted (Drewry and Macarron, 2010, Makley and Gestwicki, 2013, Wells and McClendon, 2007, Edfeldt et al., 2011).

Drug discovery has evolved in the last decades and within this realm, structure-based drug design (SBDD) (Blundell, 1996) and fragment-based lead discovery (FBLD) (Hajduk and Greer, 2007, Shuker et al., 1996) have been emerging as powerful methods for discovering high-affinity ligands for targeting proteins. FBLD has yielded a number of successful cases and these have proven to be useful for drug discovery, (Bollag et al., 2010, Drysdale and Brough, 2008, Nalepa et al., 2006, Woodhead et al., 2010, Zhu et al., 2010), offering indeed clinical candidates for therapeutic purposes. In order to undertake an efficient chemical space exploration for lead development, FBLD uses ‘fragments’ which are low molecular weight compounds with somehow minor complexity that interact weakly, yet efficiently, with a macromolecular target (Mashalidis et al., 2013, Rees et al., 2004). The ‘fragment’ concept has been extensively argued and several guidelines for library design and biophysical techniques for screening cascades have been proposed to broaden the scope and applications of this approach on drug discovery. By yielding suitable starting chemical matter with high ligand efficiencies (LEs) (Kuntz et al., 1999, Zorn and Wells, 2010) FBLD has the potential to aid effective targeting of less well-defined ligand binding pockets within the so-called ‘undruggable’ protein targets.

Historically FBLD and NMR spectroscopy have always been closely connected (Dalvit et al., 2003, Diercks et al., 2001, Huth et al., 2005, Pellecchia et al., 2008, Pellecchia et al., 2002, Shuker et al., 1996). The advent of structure-activity relationship (SAR) by NMR (Shuker et al., 1996) methodology brought FBLD to life, setting a new hallmark in drug discovery. Due to NMR spectroscopy's wide scope and inherent ability to detect weakly binding fragments (single digit mM K_d_s), its entanglement with FBLD has grown and appears to be stronger than any other exhibited by alternative FBLD-assisting techniques (Ludwig and Guenther, 2009, Rees et al., 2004, Roberts, 2000). NMR spectroscopy's ability to detect and characterise fragment binding has placed it in a preeminent position for qualitatively detecting if interactions are happening and where these interactions are taking place in the protein. In addition, the binding modes can also be established (Becattini and Pellecchia, 2006, Constantine et al., 2006, Guan et al., 2013). Moreover, by directly observing binding events NMR experiments are less prone to the kind of false-positive hit identification and artefacts found in other screening techniques.

NMR spectroscopy's ability to analyse structure, interactions and dynamics has been evolving in the direction of its main limitation, the size of molecular complexes (Fernández and Jahnke, 2004, Mittermaier and Kay, 2006, Pervushin et al., 1997). In this review we focus our attention on the available techniques and successful literature examples of NMR spectroscopy's contribution to structure-based targeting of multi-protein complexes. It is not our aim to be exhaustive on all the NMR methods available, instead to highlight how available tools can be used when handling large complexes. Emphasis will be made on current possibilities and how recent methodological developments are moving NMR spectroscopy in the direction of high molecular weight proteins and their complexes. Such biomolecules are prevalent players in key biological signalling processes and constitute attractive new drug targets against many diseases. Since NMR isotope-active protein expression is a requisite, we also highlight currently available methods to yield good spectra in challenging systems. The review will conclude with a discussion on available technology and new frontiers for targeting multi-protein complexes for drug discovery using both structural and dynamical information provided by NMR spectroscopy.

## 2 Existing NMR approaches for hit generation and characterisation

Theoretically, NMR spectroscopy parameters may serve to interrogate molecular interactions and binding. Sensitivity however limits the dynamic range of NMR observables and only a subset of such parameters is routinely monitored. Changes in chemical-shift, nuclear overhauser effects (NOEs), relaxation times or diffusion constants are often informative-rich measures acquired from NMR experiments (Hajduk et al., 2005, Harner et al., 2013, Jahnke, 2007, Lepre, 2011, Pellecchia et al., 2002). These experiments coherently identify binding events by gauging two main categories: small molecule ligand resonances or, alternatively, resonances of the protein(s) – if size of the target protein permits both ligand-based and target-based approaches can be synergistically used to provide a complete picture for an interaction. Nonetheless, NMR spectroscopy's hit generation and characterisation in lead development is still a very active field and continuously expanding (Barrett et al., 2013).

### 2.1 Ligand-based NMR

Experiments based on the resonances of the small molecule (ligand or fragment) observe only unbound ligand. Nonetheless, these are also informative of the ligand's history if their dissociation is within the timescale of these experiments (100–1000 ms). In a mixture of protein(s) and ligand(s) the effects of binding will be transferred to the unbound ligand because during the time where the ligand is bound to the protein it will not behave as a small molecule as far as NMR observables goes. During this bound period the ligand behaves like the protein – it now tumbles slowly, it portrays faster relaxation and large negative NOEs. Differences in NMR observables of ligand spectra acquired in the presence and absence of protein can therefore disclose binding (Sledź et al., 2012).

The major benefit of monitoring the ligand resonances is the absence of need to isotopically enrich the target macromolecule (Diercks et al., 2001). Additionally, the size of the macromolecular binding partner has no upper limit restriction. The binding phenomena can be observed in protein concentrations as low as 5 μM and the throughput of these experiments can be increased by cocktailing several ligands simultaneously. A common limiting step in ligand-based NMR screening is sometimes the compound's solubility in aqueous buffers and usually concentrations higher than 200 μM are a requisite for successful NMR method application.

**Fig. 1:**
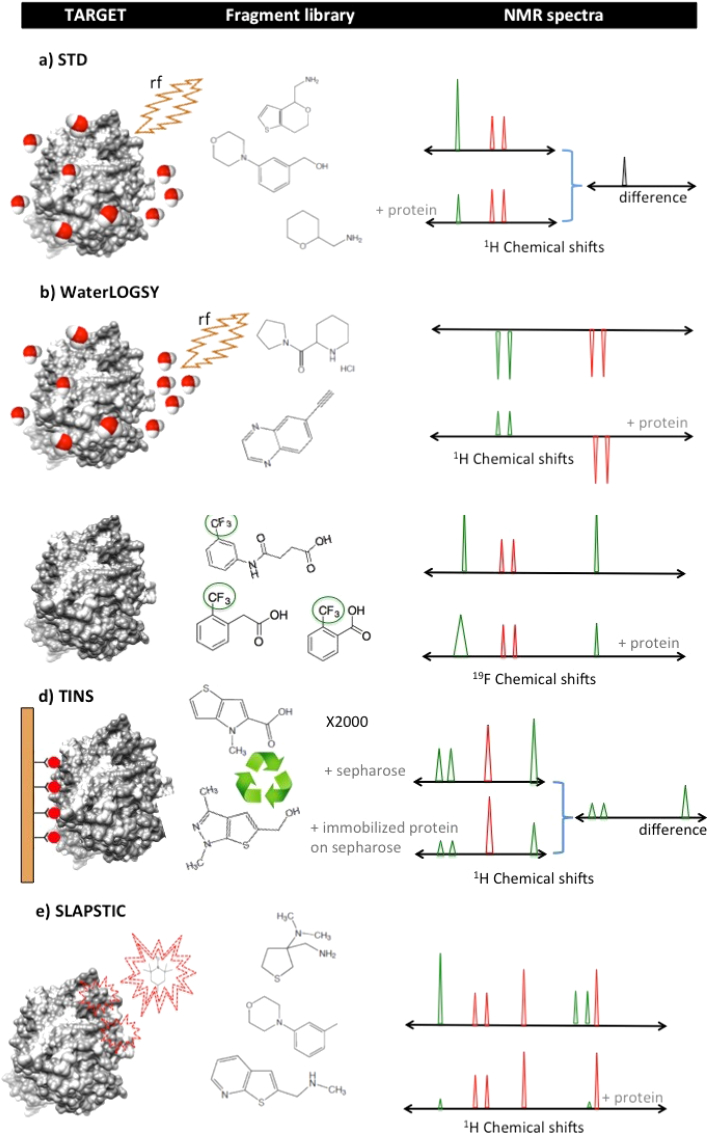
Representative NMR techniques used for hit generation. The left panel depicts the protein and the principle of the technique, the middle panel shows examples of fragment structures from the screening library that can be analysed by each technique, and the right panel shows the different spectral behaviour for hits (green resonances) and non-binders (red resonances).

Ligand-based screening techniques are extensively reviewed. Some techniques are more popular and widely used than others. Transfer NOE-type experiments such as saturation transfer difference (STD) (Mayer and Meyer, 2001, Mayer and Meyer, 1999) and water ligand-observed spectroscopy (WaterLOGSY) (Dalvit et al., 2000) occupy the top of the list for two of the most widely applied NMR techniques in hit generation (Fig. 1a, b, respectively). Both methods are capable of detecting weak interactions and the bigger the target the larger the difference between the NOE strength and sign, depicting clear binding profiles much better. While STD experiments saturate protein resonances directly, WaterLOGSY relies on saturation transfer from bulk water to any bound ligand directly or indirectly via protein by intermolecular spin diffusion.

Ligand-based NMR binding assays have proven to be robust hit generation methodologies and the current available list features not only ^1^H schemes but also heteronuclear detection methodologies (^19^F or ^31^P). In these cases, the resulting spectra are much simpler and easy to interpret and these techniques are gaining popularity in fragment screening campaigns. One such example is the binding assay known as fluorine chemical shift anisotropy and exchange for screening (FAXS) (Dalvit et al., 2003, Dalvit and Vulpetti, 2012) (Fig. 1c). Here, the observed ^19^F transverse relaxation R_2_ in the absence and presence of the protein are indicative of an interaction. Although the detection increases by working at stronger magnetic fields this method is validated in fields as low as 400 MHz. The set-up requirements make this technique suitable for wide use and benefiting from high sensitivity FAXS can reliably detect fragments starting at very low affinity.

Protein requirements for screening and hit generation are usually low for NMR techniques such as target immobilized NMR screening (TINS) (Vanwetswinkel et al., 2005) (Fig. 1d) which allows using the same sample for more than 2000 fragments. This method therefore reduces significantly the amount of target protein to be used in a screening campaign and exhibits a throughput of 1500 compounds per 4 days. The TINS approach has been shown to generate and validate hits on a variety of targets, making it a very attractive option to start a hit discovery process. Another alternative to reduce the protein requirements is using spin-labels. Spin labels attached to protein side chain as a tool to identify interacting compounds (SLAPSTIC) (Jahnke, 2002, Jahnke et al., 2001) (Fig 1e) requires selective labelling of the target residues (e.g. lysine, tyrosine, cysteine, histidine or methionine) or the use of a paramagnetically-tagged known binding ligand. As a requirement at least one residue should be as close as possible to the binding site, but without being involved in the interaction. The paramagnetic relaxation rates are higher than the diamagnetic rates on a given target, and this allows a reliable detection of hits as low as 50 times less protein. Moreover, the discrimination of hits and non-hits becomes so evident that it is possible to automate the analysis of SLAPSTIC experiments.

### 2.2 Protein-based NMR

Protein resonances are information-rich NMR observables that can be used to reveal structure, function and dynamics under most circumstances. With the possibility to study both solution and solid-state samples NMR offers one of the most robust methods for detecting and characterising physical interactions. In FBLD it is well documented how useful this data can be. Direct assessment of protein resonances unveils where on the protein an interaction may occur and also how this interaction is made.

Protein-based NMR methods historically gave birth to fragment-based screening approaches. In order to map which specific residues in the sequence, and ultimately the structure, are involved in the interaction, a high level of backbone amide resonance assignment is necessary. This requires double isotope-labelling and additional experimentation, usually from triple-resonance approaches. Although there are restrictions imposed by the target's molecular weight (around 40 kDa) nowadays there are several relaxation-optimized NMR techniques to tackle the relaxation and linewidth problems accompanying high molecular weight targets. Additional improvements on the experimental time to undertake these types of experiments have been making NMR a high throughput approach strengthening its position in hit generation and characterization.

The most common protein-based NMR technique is the 2D ^1^H–^15^N heteronuclear single quantum correlation (HSQC) (Bodenhausen and Ruben, 1980) of ^15^N-labelled proteins. Since this experiment observes the amide NH groups, which become chemically unique in the context of the protein's tertiary structure, it allows monitoring binding events in a residue specific manner. Using 2D HSQC one can collect the spectra in the absence and in the presence of small-molecule compounds or other target proteins. This simple approach allows obtaining chemical shift perturbation (CSP) (Williamson, 2013) data. A comparison of 2D ^1^H–^15^N HSQC's in the presence and absence of the putative interaction partner discloses binding events. These can be monitored by following the shifting residues (when in a fast exchange regime) or decrease in intensity (and may be even disappearance and appearance again) if in a slow exchange regime. Perturbed residues are usually identified as those which exhibit changes beyond one or two standard deviations. If the protein structure is known, it is possible to immediately identify the binding site by mapping these residues onto the protein structure. In order to eliminate the possibly biased cut-off to set when analysing CSPs, one can use SAMPLEX. (Krzeminski et al., 2010) Such an approach guarantees a much more unbiased selection of the relevant CSPs by automating this selection and predicting statistically significant residues that should have shown CSPs (either because of experimental errors or because they are prolines), based on the three-dimensional structure of the target.

**Fig. 2:**
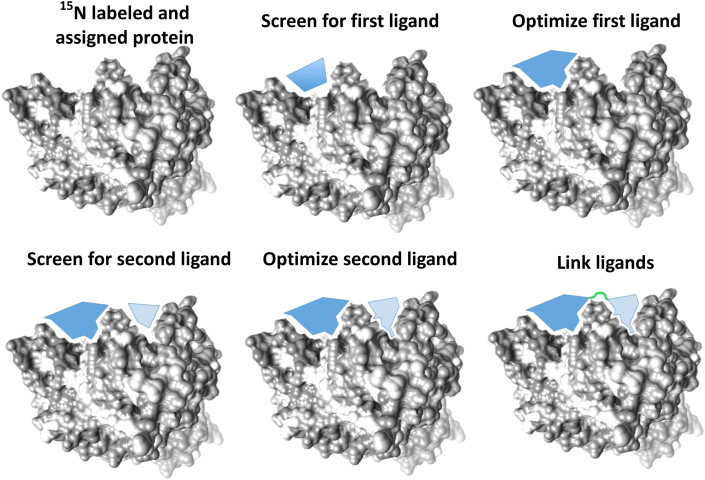
Schematic representation of the stepwise approach of SAR by NMR.

The chemical shift perturbation assay has been used extensively to identify binding sites of small molecules. This approach gained even more popularity when SAR by NMR was introduced in 1996 (Shuker et al., 1996). SAR by NMR (Fig. 2) first uses CSP data from weakly interacting compounds in order to optimize them for a given site in the protein. The second step is to find adjacent sites in the protein where another weakly interacting compound is binding and optimize it as far as possible. The third step is to disclose the orientation of the bound ligands in order to guide their linkage and elaboration and maintain this orientation in the final compound, thereby achieving high specificity to that target. This technique therefore allows high-affinity ligand elaboration and reduces the laborious chemical synthesis necessary for such potency.

The SAR by NMR method has facilitated the development of highly potent and specific compounds and it continues to be one of the most popular and successful NMR technique for FBLD (Hajduk et al., 1999, Hajduk et al., 2000, Oost et al., 2004, Petros et al., 2010, Sun et al., 2012).

### 2.3 Ligand-bound conformation and protein binding sites identification by NMR

Protein and protein-small molecule structure determination features on many NMR techniques, and this allows binding site identification by mapping the CSPs obtained upon small molecule titration. However its use is limited to small and medium sized protein, as it requires backbone and side chain assignment for structure calculation. Being also a time consuming duty, several amenable techniques have been contributing to structural information on protein-small molecule complexes. This information is extremely useful to guide further medicinal chemistry hit-lead optimization campaigns.

**Fig. 3:**
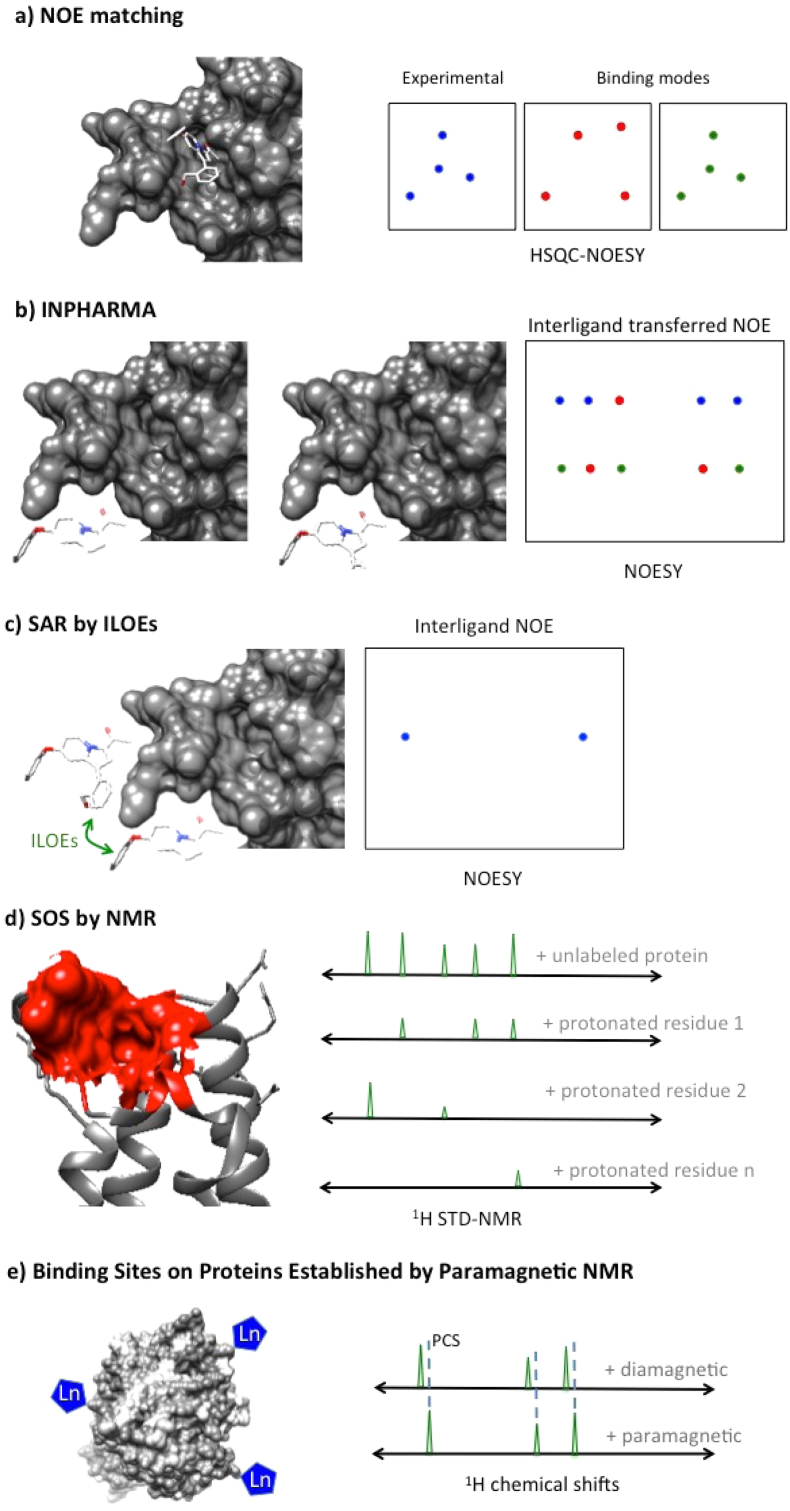
NMR techniques used to study ligand-bound conformation and protein binding sites identification. The left panel depicts the protein and the necessary manipulations for each method. The right panel shows the typical spectra for these techniques.

Of particular interest are techniques that do not require protein resonance assignments to obtain structural information. The majority of these NMR techniques use some intermolecular NOEs to generate reasonably good protein-small molecule complex models. NOE matching (Fig. 3a) (Constantine et al., 2006) is one such method and it is one of the most popular methods aiding drug design currently. This NMR technique uses ^13^C-edited and ^13^C/^15^N-filtered HSQC-NOESY spectra to evaluate small molecule binding poses. Here only ^1^H assignments of the bound small molecule are critical and by comparing the experimental above-mentioned spectra with predicted ones (e.g. from binding poses obtained by computational docking) it is possible to identify the matches. This matching is then scored and if sufficient protein-small molecule NOEs can be measured, informative-rich models can be crafted.

Other popular approaches, like STD and WaterLOGSY-based ones, also can illustrate small molecule binding mode knowledge to some extent, by performing careful group epitope mapping (GEM) around the ligand resonances. However the most robust and widely used method is the so-called INPHARMA (Fig. 3b) (Sanchez-Pedregal et al., 2005). The INPHARMA method can be used to obtain relative binding orientations between two ligands that compete for the same binding pocket on the protein target. Based on the binding mode of one ligand the competitive ligand's binding epitope are readily discriminated. Atoms from different small molecules interacting with the same protein residues will manifest the strongest INPHARMA inter-ligand NOEs and consequently, relative binding orientations can be derived.

When multiple hits are found for a given protein there is an opportunity to exploit the theoretical contribution from the linking coefficient (Borsi et al., 2010, Jencks, 1982, Murray and Verdonk, 2002) to enhance the tethered and validated neighbouring small molecules into a more potent lead-like compound. This reward is predominantly due to an entropic gain on tethering adjacent site binders, which will translate in higher affinity for the binding site. The structure activity relationships by interligand nuclear Overhauser effect (SAR by ILOEs; Fig. 3c) (Becattini and Pellecchia, 2006) is a 2D NOESY NMR experiment that can be used to detect when two small molecule ligands bind to a protein next to one another. The method can also allow the determination of the small molecules binding orientation relative to each other. Only ligands that bind in relatively close proximity to each other will generate a signal, allowing identification of fragments that may be productively linked to generate higher affinity bidentate compounds. Another advantage of the ILOE technique is that it can be applied to proteins that are too large to study by protein-observed NMR methods, and even in the absence of the protein structure. As with many NMR and other biophysical techniques, ILOE may be prone to compound and protein aggregation artifacts that should be carefully taken into account when interpreting the results (Sledz et al., 2010).

Other NMR based methods are also capable of identifying protein binding sites both using isotopically labelled or natural abundance proteins. The ability to identify a binding site using structural information obtained by Overhauser effects and selective labelling (SOS; Fig. 3d) (Hajduk et al., 2004) is a great alternative when X-ray or commonly-used NMR methods for structure-based lead design are not possible. The SOS-NMR method uses STD NMR spectroscopy on uniformly deuterated samples except on some specific residues. Monitoring the STD spectra on these specific and differently labelled samples, the composition of the binding site can be obtained. If three-dimensional information of the protein is available the identification of the binding site is straightforward but even without this information this approach can contribute to a better rationale to guide later optimization of the small molecule ligand. This can be achieved by targeting some of the key residues present in the SOS-NMR revealed binding site.

A relevant approach for initial small molecule validation, both mapping its binding site and its binding pose, was recently introduced (Guan et al., 2013). This paramagnetic NMR method is based on pseudocontact shifts (PCSs) that are used to derive intermolecular structural restraints (Fig. 3e). This method requires the attachment of relatively rigid paramagnetic agent-loaded tags onto the protein of interest. However, once this is achieved and the integrity and activity of the modified protein target is verified, small molecule PCSs can be obtained from 1D ^1^H spectra to be used as docking restraints. Structural information of the protein is indispensable to obtain the three-dimensional position of the target's binding site. This promising approach can be applied to non-isotopically labelled proteins, making it very attractive for hit validation in fragment screening.

## 3 Handling large multi-protein complexes: experiences and lessons using NMR

Large molecular weight targets including multi-subunit protein complexes exhibit a major limitation both to X-ray and NMR structural data accomplishment. The application of NMR methods to high MW proteins is limited primarily by the fact that the linewidths increase with MW as larger molecules have longer rotational correlation times and as a result much shorter transverse relaxation times (T_2_). In hit-to-lead optimization campaigns structural data is the limiting step to guide a rationale for compound elaboration and introducing chemical modifications. Knowledge of the target protein's structure doesn't imply that the binding of a small molecule hit will be easily validated structurally. Even if the target protein can be crystallized, soaking sometimes is not possible and this would mean that co-crystallization and new crystallization conditions have to be re-iterated. These requirements may be particularly challenging for multisubunit protein complexes.

In order to use NMR as a complementary technique to provide means to structural information at atomic resolution for these types of targets, NMR active isotope labelling is necessary. Size remains the main limitation, however multiple labelling schemes and experiments are readily available, making it now possible to handle assemblies as big as the 1 mega Dalton proteasome complex (Griswold and Dahlquist, 2002, Mainz et al., 2013).

### 3.1 Isotopic labelling strategies

Over recent years several labelling strategies have been introduced, helping NMR structural knowledge to be accessible on large molecular weight targets, multi-domain protein or multi-protein complexes (homo or heteromeric both symmetrical or asymmetrical).

**Fig. 4:**
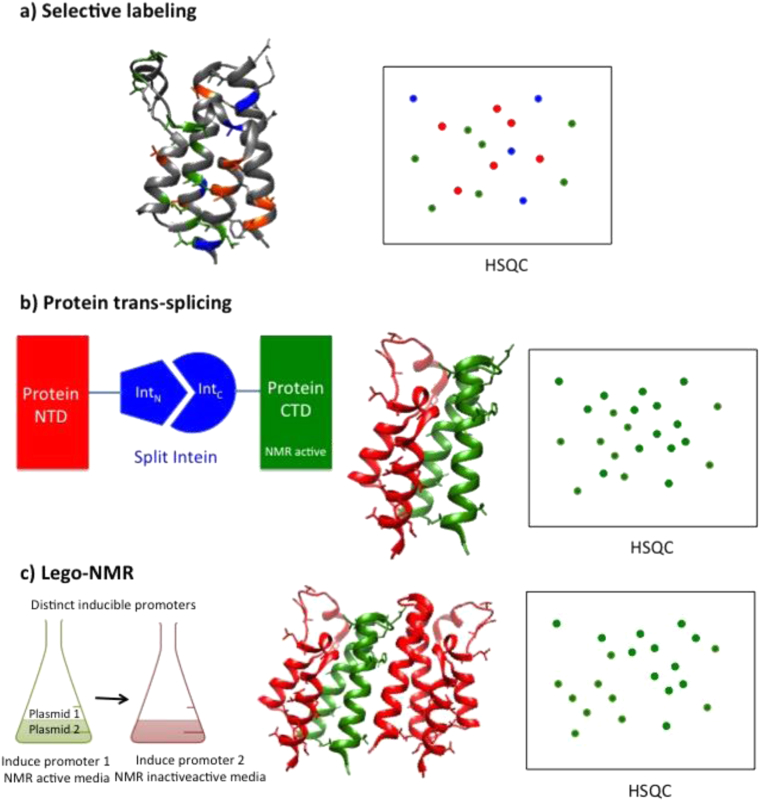
Isotopic labelling strategies to aid NMR studies of multi-protein targets. (a) Selective labelling can be done separately (e.g one aminoacid at a time, shown in blue, red and green) allowing residue-specific information if a set of residues is labelled. (b) Protein trans-splicing method where split inteines catalyse the NMR-active and NMR-inactive protein ligation (c) Lego-NMR uses different inducible promoters to form a multiprotein complex within the same host. Using this constructs different subunits can be isotopically labelled allowing multi-protein complex handling by NMR.

When handling large molecular weight targets (>50 kDa) selective labelling (Fig. 4a) is a good option to decrease resonance overlap on heteronuclear spectra. This is possible by adding just the desired residues labelled isotopically in the expression media (Ayala et al., 2009, Tugarinov and Kay, 2004). Although some scrambling can occur a number of residues (e. g Alanine, Methionine, Isoleucine, Threonine, Leucine and Valine) were found to be good reporters for binding and interaction sites (Guo and Tugarinov, 2009, Ollerenshaw and Tugarinov, 2003, Tugarinov and Kay, 2004, Tugarinov et al., 2005, Tugarinov et al., 2004, Velyvis et al., 2009).

The concept of segmental isotopic labelling of multi-domain and fusion proteins is a robust alternative to obtain NMR spectra (Busche et al., 2009, Cowburn et al., 2004, Muona et al., 2010). This approach allows the selective labelling in just a portion of the target protein and it has been demonstrated to multi-domain and fusion proteins via protein trans-splicing (PTS) (Mootz, 2009, Muona et al., 2010) using split DNAe intein. PTS is an auto-catalytic process that works by yielding an active form of the split intein which catalyses the protein ligation. This works without the need for refolding protocols or additional residues or modifications present in other segmental labelling alternatives. PTS was validated both *in vitro* and in cells by using a time-delayed dual-expression system to achieve effective labelling (Fig. 4b).

**Fig. 5:**
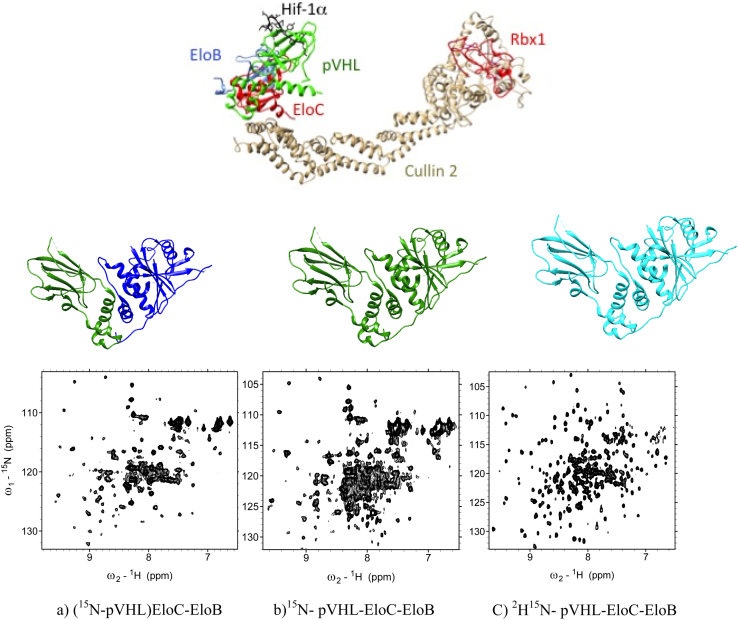
Different labelling schemes and respective spectra in von Hippel-Lindau protein (pVHL) E3 ubiquitin ligase multi-subunit complex. The top panel shows a model for the E3 complex containing pVHL-EloC-EloB-Cullin2-Rbx1 subunits and bound peptide from substrate Hif-1a. The lower panel shows spectra for the trimeric complex pVHL-EloC-EloB with pVHL subunit only labelled with ^15^N (a); and the full complex labelled with ^15^N (b) and also perdeuterated (c).

Most of the multi-protein complexes are heteromeric assemblies of more than one protein and co-expression of all the components usually result in spectral crowding. This loss of detail impedes the required detail for structural examination and small molecule validation. A significant drop in yield can occur when performing *in vitro* reconstitution of the complex from separately expressed and co-lysed NMR active and NMR inactive subunits (Fig. 5a). This is particularly relevant to individual subunits that are not stable unless in complex with the partner subunit and therefore do require co-expression to achieve stable and soluble protein complex (Van Molle et al., 2012). A novel approach termed LEGO-NMR (Mund et al., 2013) circumvents these limitations by using induced promoters that are switched on and off at different stages. This tight regulation is accomplished by having different inducers and repressors, which prevent leaky expression in both the active and inactive NMR media (Fig. 4c).

Most importantly all of these approaches preserve the possibility to carry out sequential resonance assignments by triple-resonance NMR experiments.

### 3.2 Water(s) and NMR

In multi-protein complexes or PPIs, water is often present and plays an important role in driving and stabilizing the interactions. Water molecules can be displaced from the interaction surface or by acting on the folding transition upon complex assembly. NMR can address these active roles of waters both qualitatively and quantitatively.

One of the most standard approaches to measure water-backbone proton exchange is the CLEAN chemical exchange (CLEANEX) experiment (Hwang et al., 1998, Hwang et al., 1997). The phase-modulated version of CLEANEX is relatively artefact-free allowing accurate measurements of exchange rates between water and amide NH protons by magnetization transfer. The experiment is executed in a difference manner, one spectrum with and another without water inversion. CLEANEX difference spectra are selective only to magnetization that is transferred from water protons to protein amide protons. In this way, easy and qualitative interpretation of the spectra is generally obtained. On the other hand, effective exchange rates can be obtained by varying the mixing time in the experiment.

The other face of the coin in water and NMR is water's contribution to the decreasing of spectral crowding. Experiments are available which explore the concept that, for binding studies, solvent exposed residues are most influential. Being solvent accessible these residues can act as reporters and allow the study of intermolecular interactions. Whereas buried residues will most likely not contribute directly for such binding events. This significantly decreases the number of residues in the spectra but still permits following of binding events effectively. Solvent exposed amide (SEA) techniques (Lin et al., 2002, Pellecchia et al., 2001) have been used to study both the local folding/unfolding kinetics and protein energetic stability. Moreover, rapidly exchanging protons reveal information about H-bonding, surface dynamics and allostery, all informative rich parameters to characterise binding events.

### 3.3 Exploring multi-protein complexes and protein–protein interactions with NMR

Using NMR spectroscopy to explore multi-protein complexes and PPIs is well known to be a laborious task (Bonvin et al., 2005) and over the years many techniques have been introduced to simplify this task (Fry, 2006, Fuller et al., 2009, Jubb et al., 2012). It is hard to think of a greater hallmark to high molecular weight NMR handling experiment than transverse relaxation-optimised spectroscopy (TROSY) (Pervushin et al., 1997). With their introduction TROSY-based methods are established as the most preeminent NMR approaches presently available (Fig. 5c). By suppressing transverse relaxation in multidimensional NMR experiments, TROSY has reduced linewidths for every NMR-active target studied. Transverse relaxation increases significantly with the molecular weight of proteins. TROSY experiments are based on the constructive use of interference between the main ^1^H, ^15^N and ^13^C transverse relaxation rates, dipole–dipole (DD) coupling and chemical shift anisotropy (CSA). Because these relaxation rates present a molecular size-independent ratio, a comparable reduction of the overall transverse relaxation rates can be expected for larger proteins. It is theoreticized that cancelation of both these pathways is optimal around 1000 MHz ^1^H frequencies. Upon decoupling a total cancelation of transverse relaxation effects within a ^15^N–^1^H moiety can be achieved for one of the four multiplet components. The narrow component observed in this experiment will just have the residual linewidth coming from DD interactions, which can be further suppressed with perdeuteration (Sattler and Fesik, 1996).

Inclusion of ^2^H in proteins has an important impact by reducing magnetization through the spin system of dipolar-coupled protons (spin diffusion). Using this incorporation to decrease the proton density in proteins also eradicates spin diffusion pathways, improving signal-to-noise in the NMR spectra. Additionally, this incorporation allows identification of PPI sites. In an elegant cross-saturation TROSY-based experiment (Takahashi et al., 2000), a ^2^H–^15^N uniformly labelled protein is complexed with a non-labelled interacting protein; taking advantage of higher proton density, aliphatic protons are irradiated using a radio frequency. This allows for spin diffusion within the non-labelled protein to happen instantly throughout the protein, whereas the double-labelled protein will remain unaffected. If both proteins interact it is expected that cross-saturation transfer to occur only at the residues in the protein–protein interface. Simply by measuring the disappearing residues high-resolution information on the interface can be mapped into the protein.

Knowledge about interacting protein interfaces is extremely useful if one is targeting these interactions for disruption or stabilization e.g. using a small molecule (Buckley et al., 2012a, Buckley et al., 2012b, Thiel et al., 2012, Van Molle et al., 2012). Usually this targeting is hindered by lack of well-defined pockets or grooves on PPIs binding sites, which figure a rather more flat character with a relatively large contact area. Inherently lower LEs are found in PPI-targeting small molecules and to gain selectivity and affinity more than one ‘binding subsite’ usually needs to be targeted with the small molecule. The correlation between successfully targeted PPIs, hit rates on PPIs and PPI-focused fragment library to PPIs are discussed to lead the way to successful targeting (Ciulli et al., 2006, Gretes et al., 2009, Kumari and van der Hoorn, 2011, Mortenson and Murray, 2011, Stukalov et al., 2012).

PPIs ability to bind small molecules (ligandability) is usually lower compared to that observed e.g. at buried enzyme active sites (Edfeldt et al., 2011, Surade and Blundell, 2012, Van Molle et al., 2012). However NMR screening can deal with this low LE small molecules, by detecting initial high mM dissociation constant hits. With a constant paradigm shift, happening both in academy and industry, in our perspective on what are the most relevant targets on which to focus drug discovery efforts, it becomes important that target ligandability is assessed in the best possible way. By optimizing protein and ligand concentration, hit rate can be fine-tuned to yield true positive hits, which can be structurally validated (Dias et al., 2014). Failure on detecting weak but true hits by some biophysical techniques i.e. false negatives can often genuinely be happening in this category of targets. NMR based screening's ability to grasp these starting compounds allows unique opportunities to succeed in a FBLD campaign.

### 3.4 Dynamics and NMR

**Fig. 6:**
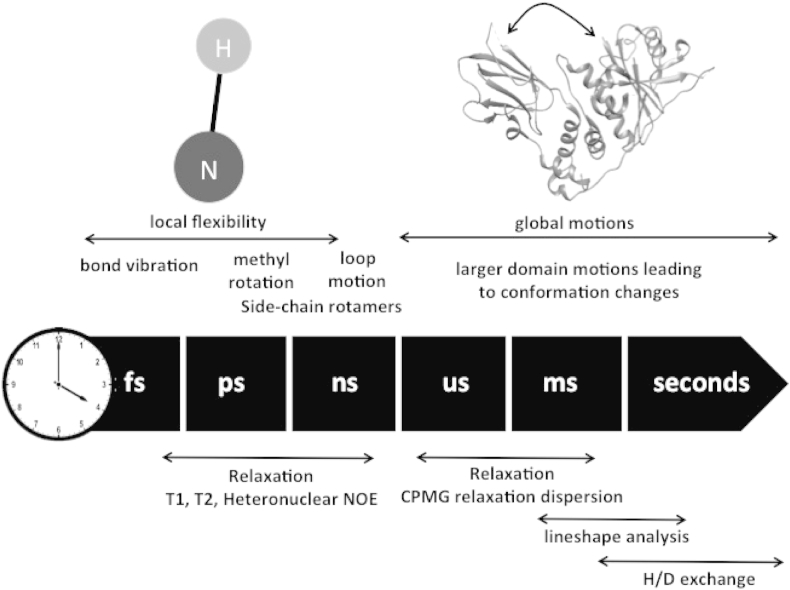
Different protein dynamics and time scales accessible to NMR spectroscopy.

It is well recognized that solution NMR spectroscopy is a powerful method to study both structural and dynamic properties of proteins (Fig. 6). The quantitative information of the length and time scales of protein's internal motions allows unique understanding of their biological function (Kern and Zuiderweg, 2003, Palmer, 2004, Wang et al., 2001). This insight offers knowledge on protein-ligand (Long et al., 2013) and protein–protein interactions (Li et al., 2013); as well as insights into allosteric mechanisms and by providing a baseline for molecular dynamic trajectories (Showalter and Brüschweiler, 2007).

Obtaining dynamics parameters from protein backbone ^15^N relaxation measurements (Kay et al., 1989) has been a common experimental practice over the past few decades. Albeit well-described, some systematic errors are reported, especially for perdeuterated proteins and when using TROSY (Lakomek et al., 2012). Nonetheless, this information is extremely useful to describe picosecond-nanosecond bond vectors motion. The main techniques routinely used focus on ^15^N spin relaxation (longitudinal T_1_, transverse T_2_ and steady-state ^1^H–^15^N heteronuclear NOE) by gauging the reorientation of ^15^N–H bond vectors allowing the study of backbone dynamics and also every ^15^N-containing side chains. This relaxation data is of great value and it informs the rate of overall molecular tumbling (also known as τ_c_ – particularly accurate in the case of isotropic molecular tumbling) or the components of the diffusion tensor in the case of anisotropic overall motion. By comparing fluctuations in local magnetic field that lead to relaxation on the full-length molecule versus residue-specific relaxation it is possible to inform on the protein's local internal motion. A parameter describing the amplitude of internal motions (S^2^) (Lipari and Szabo, 1982) and effective rate constant for internal dynamics can also be readily obtained. This data allows a protein mapping according to its inherent propensity to exhibit dynamic behaviour in this timescale and therefore inform on both sample stability and binding events.

Notwithstanding, events that cause reasonably high amplitude molecular motions, such as direct protein or ligand interaction or allosteric regulation, are most likely to fall in the microsecond to millisecond timescale. Carr-Purcell-Meiboom-Gill (CPMG) relaxation dispersion (RD) (Mulder et al., 2001, Neudecker et al., 2009, Vallurupalli et al., 2011) NMR experiments were developed to tackle these scenarios. These experiments have been brilliantly applied to protein folding, mostly considering fractional populated states and transition-state theory yielding both kinetic and structural information in previously ‘invisible’ protein states (Baldwin and Kay, 2009, Hansen et al., 2009, Kay, 1998). It is proposed that all proteins conformations pre-exist to different extents in solution and the interactions occur by a conformational selection (Boehr et al., 2009) mechanism. Among other NMR observables, CPMG-RD allows the characterization of these substates. Measurements of chemical exchange broadening can support models beyond the induced-fit hypothesis, for interactions or conformational changes in which ligand binding or post-translational modifications may select one conformation from a pre-existing ensemble.

Interrogation of biomolecular binding events endowing conformational changes has been reported and the role of pre-existing equilibria of multiple conformational intermediates can be interrogated using NMR. The role of dynamics as an entropic carrier of free energy of allostery (Palmer, 2004) has been inspected and it has been suggested that extensive enthalpy/entropy exchange usually happens during the formation of a protein–protein interface (Kern and Zuiderweg, 2003, Stone, 2001). Understanding the fundamentals of conformational entropy of proteins (Boehr et al., 2009, Frederick et al., 2007, Tzeng and Kalodimos, 2012) will better inform the elucidation of protein activity and has the potential to lead to better protein-directed pharmaceuticals.

## 4 Discussion and new frontiers

NMR spectroscopy is now a well-established technique to elucidate the structure, interaction and dynamics of molecules in solution and to guide structure-based lead discovery approaches.

Over the last decade a clear trend has been emerging in the realization that many of the most attractive drug targets are likely to be of large size and complexity. Depicting inherently more complex structures than those historically investigated (e.g. catalytically-active fragments or single domains of individual proteins), these macromolecular assemblies have proven to be demanding targets to modern structural biology. The possibility to handle large molecular weight biomolecules is however now becoming a reality and it is being diffused to more and more biological systems. Crucially, it is slowly gaining momentum into drug discovery projects too. PPIs often fall within this category of challenging and complex systems, and as a result it is not straightforward to make progress on them using conventional approaches. The combination of topological features e.g. flat, extended and often highly hydrated binding sites bearing few small pockets, and low ligandability characteristics of PPIs limit their profiteering. Nonetheless, successful campaigns and recent literature examples have established the high-risk/high-reward status of many PPIs. With the new paradigm of targeting PPIs in drug discovery, we predict that NMR approaches will continue to play a crucial role in facilitating traction against these targets. This requires studying proteins and obtaining structural information beyond what has been traditionally considered as one of the main limitations of NMR, i.e. the size of the molecular target.

Understanding what governs protein dynamics in a three-dimensional manner is still a challenging task. However NMR spectroscopy has been evolving to better address both protein dynamics and structure in difficult systems. This has included the development of ingenious labelling schemes and new pulse sequences. Amongst these, we anticipate that TROSY and CPMG-RD will hold the most promise and potential at pushing the frontiers of NMR in SBDD. Furthermore, Methyl-TROSY should be considered as a probing technique to help bringing high molecular weight targets in focus for FBLD and compound screening, something that isn't done routinely.

In our opinion, with the technology we have in hand and prospective developments expected in future years, we believe that the inclusion of millisecond timescale dynamics information provided by NMR will aid targeting and development of high-quality chemical probes against multi-protein complexes. This information could present structural data on allosteric intermediates and transient binding sites so that these could then be discovered and targeted more efficiently. It is well documented that these mechanisms exist, especially in multi-protein complexes however we still seem to be one small step away from their structural comprehension and further usage in lead design. It is well understood that protein conformational dynamics is encoded in their sequence and structure, and that it is a fundamental requisite for their biological function (Karplus and Kuriyan, 2005). In moving targets forward within a given drug discovery pipeline, we predict that a triad of Structure-Dynamics-Targeting would allow manipulating protein function with increased specificity and potency.

## References

[bib1] Ayala I., Sounier R., Usé N., Gans P., Boisbouvier J. (2009). An efficient protocol for the complete incorporation of methyl-protonated alanine in perdeuterated protein. J. Biomol. NMR.

[bib2] Baldwin A.J., Kay L.E. (2009). NMR spectroscopy brings invisible protein states into focus. Nat. Chem. Biol..

[bib3] Barrett P.J., Chen J., Cho M.-K., Kim J.-H., Lu Z., Mathew S., Peng D., Song Y., Van Horn W.D., Zhuang T., Sönnichsen F.D., Sanders C.R. (2013). The quiet renaissance of protein nuclear magnetic resonance. Biochemistry.

[bib4] Becattini B., Pellecchia M. (2006). SAR by ILOEs: an NMR-based approach to reverse chemical genetics. Chemistry.

[bib5] Blundell T.L. (1996). Structure-based drug design. Nature.

[bib6] Bodenhausen G., Ruben D.J. (1980). Natural abundance nitrogen-15 NMR by enhanced heteronuclear spectroscopy. Chem. Phys. Lett..

[bib7] Boehr D.D., Nussinov R., Wright P.E. (2009). The role of dynamic conformational ensembles in biomolecular recognition. Nat. Chem. Biol..

[bib8] Bollag G., Hirth P., Tsai J., Zhang J., Ibrahim P.N., Cho H., Spevak W., Zhang C., Zhang Y., Habets G., Burton E.A., Wong B., Tsang G., West B.L., Powell B., Shellooe R., Marimuthu A., Nguyen H., Zhang K.Y.J., Artis D.R., Schlessinger J., Su F., Higgins B., Iyer R., D'Andrea K., Koehler A., Stumm M., Lin P.S., Lee R.J., Grippo J., Puzanov I., Kim K.B., Ribas A., McArthur G.A., Sosman J.A., Chapman P.B., Flaherty K.T., Xu X., Nathanson K.L., Nolop K. (2010). Clinical efficacy of a RAF inhibitor needs broad target blockade in BRAF-mutant melanoma. Nature.

[bib9] Bonvin A.M., Boelens R., Kaptein R. (2005). NMR analysis of protein interactions. Curr. Opin. Chem. Biol..

[bib10] Borsi V., Calderone V., Fragai M., Luchinat C., Sarti N. (2010). Entropic contribution to the linking coefficient in fragment based drug design: a case study. J. Med. Chem..

[bib11] Buckley D.L., Gustafson J.L., Van Molle I., Roth A.G., Tae H.S., Gareiss P.C., Jorgensen W.L., Ciulli A., Crews C.M. (2012). Small-molecule inhibitors of the interaction between the E3 Ligase VHL and HIF1α. Angew. Chem. Int. Ed..

[bib12] Buckley D.L., Van Molle I., Gareiss P.C., Tae H.S., Michel J., Noblin D.J., Jorgensen W.L., Ciulli A., Crews C.M. (2012). Targeting the von Hippel–Lindau E3 ubiquitin ligase using small molecules to disrupt the VHL/HIF-1α interaction. J. Am. Chem. Soc..

[bib13] Busche A.E.L., Aranko A.S., Talebzadeh-Farooji M., Bernhard F., Dötsch V., Iwaï H. (2009). Segmental isotopic labeling of a central domain in a multidomain protein by protein trans-splicing using only one robust DnaE intein. Angew. Chem. Int. Ed..

[bib14] Ciulli A., Williams G., Smith A.G., Blundell T.L., Abell C. (2006). Probing hot spots at protein−ligand binding sites: a fragment-based approach using biophysical methods. J. Med. Chem..

[bib15] Constantine K.L., Davis M.E., Metzler W.J., Mueller L., Claus B.L. (2006). Protein-ligand NOE matching: a high-throughput method for binding pose evaluation that does not require protein NMR resonance assignments. J. Am. Chem. Soc..

[bib16] Cowburn D., Shekhtman A., Xu R., Ottesen J.J., Muir T.W. (2004). Segmental isotopic labeling for structural biological applications of NMR. Methods Mol. Biol..

[bib17] Cummings C.G., Hamilton A.D. (2010). Disrupting protein–protein interactions with non-peptidic, small molecule α-helix mimetics. Curr. Opin. Chem. Biol..

[bib18] Dalvit C., Vulpetti A. (2012). Technical and practical aspects of ^19^F NMR-based screening: toward sensitive high-throughput screening with rapid deconvolution. Magn. Reson. Chem..

[bib19] Dalvit C., Pevarello P., Tatò M., Veronesi M., Vulpetti A., Sundström M. (2000). Identification of compounds with binding affinity to proteins via magnetization transfer from bulk water. J. Biomol. NMR.

[bib20] Dalvit C., Fagerness P.E., Hadden D.T.A., Sarver R.W., Stockman B.J. (2003). Fluorine-NMR experiments for high-throughput screening: theoretical aspects, practical considerations, and range of applicability. J. Am. Chem. Soc..

[bib21] Dias D.M., Van Molle I., Baud M.G.J., Galdeano C., Geraldes C.F.G.C., Ciulli A. (2014). Is NMR fragment screening fine-tuned to assess druggability of protein–protein interactions?. ACS Med. Chem. Lett..

[bib22] Diercks T., Coles M., Kessler H. (2001). Applications of NMR in drug discovery. Curr. Opin. Chem. Biol..

[bib23] Drewry D.H., Macarron R. (2010). Enhancements of screening collections to address areas of unmet medical need: an industry perspective. Curr. Opin. Chem. Biol..

[bib24] Drysdale M., Brough P. (2008). Medicinal chemistry of Hsp90 inhibitors. Curr. Top. Med. Chem..

[bib25] Edfeldt F.N.B., Folmer R.H.A., Breeze A.L. (2011). Fragment screening to predict druggability (ligandability) and lead discovery success. Drug. Discov. Today.

[bib26] Fernández C., Jahnke W. (2004). New approaches for NMR screening in drug discovery. Drug. Discov. Today.

[bib27] Frederick K.K., Marlow M.S., Valentine K.G., Wand A.J. (2007). Conformational entropy in molecular recognition by proteins. Nature.

[bib28] Fry D.C. (2006). Protein–protein interactions as targets for small molecule drug discovery. Biopolymers.

[bib29] Fuller J.C., Burgoyne N.J., Jackson R.M. (2009). Predicting druggable binding sites at the protein–protein interface. Drug. Discov. Today.

[bib30] Gretes M., Lim D.C., de Castro L., Jensen S.E., Kang S.G., Lee K.J., Strynadka N.C.J. (2009). Insights into positive and negative requirements for protein–protein interactions by crystallographic analysis of the β-lactamase inhibitory proteins BLIP, BLIP-I, and BLP. J. Mol. Biol..

[bib31] Griswold I.J., Dahlquist F.W. (2002). Bigger is better: megadalton protein NMR in solution. Nat. Struct. Biol..

[bib32] Guan J.-Y., Keizers P.H.J., Liu W.-M., Löhr F., Skinner S.P., Heeneman E.A., Schwalbe H., Ubbink M., Siegal G. (2013). Small-molecule binding sites on proteins established by paramagnetic NMR spectroscopy. J. Am. Chem. Soc..

[bib33] Guo C., Tugarinov V. (2009). Identification of HN-methyl NOEs in large proteins using simultaneous amide-methyl TROSY-based detection. J. Biomol. NMR.

[bib34] Hajduk P.J., Greer J. (2007). A decade of fragment-based drug design: strategic advances and lessons learned. Nat. Rev. Drug. Discov..

[bib35] Hajduk P.J., Dinges J., Schkeryantz J.M., Janowick D., Kaminski M., Tufano M., Augeri D.J., Petros A., Nienaber V., Zhong P., Hammond R., Coen M., Beutel B., Katz L., Fesik S.W. (1999). Novel inhibitors of Erm methyltransferases from NMR and parallel synthesis. J. Med. Chem..

[bib36] Hajduk P.J., Gomtsyan A., Didomenico S., Cowart M., Bayburt E.K., Solomon L., Severin J., Smith R., Walter K., Holzman T.F., Stewart A., McGaraughty S., Jarvis M.F., Kowaluk E.A., Fesik S.W. (2000). Design of adenosine kinase inhibitors from the NMR-based screening of fragments. J. Med. Chem..

[bib37] Hajduk P.J., Mack J.C., Olejniczak E.T., Park C., Dandliker P.J., Beutel B.A. (2004). SOS-NMR: a saturation transfer NMR-based method for determining the structures of protein−ligand complexes. J. Am. Chem. Soc..

[bib38] Hajduk P.J., Huth J.R., Fesik S.W. (2005). Druggability indices for protein targets derived from NMR-based screening data. J. Med. Chem..

[bib39] Hansen D.F., Feng H., Zhou Z., Bai Y., Kay L.E. (2009). Selective characterization of microsecond motions in proteins by NMR relaxation. J. Am. Chem. Soc..

[bib40] Harner M.J., Frank A.O., Fesik S.W. (2013). Fragment-based drug discovery using NMR spectroscopy. J. Biomol. NMR.

[bib41] Higueruelo A.P., Schreyer A., Bickerton G.R.J., Pitt W.R., Groom C.R., Blundell T.L. (2009). Atomic interactions and profile of small molecules disrupting protein-protein interfaces: the TIMBAL database. Chem. Biol. Drug. Des..

[bib42] Hopkins A.L., Groom C.R. (2002). The druggable genome. Nat. Rev. Drug. Discov..

[bib43] Huth J.R., Sun C., Sauer D.R., Hajduk P.J. (2005). Utilization of NMR-derived fragment leads in drug design. Meth. Enzymol..

[bib44] Hwang T.L., Mori S., Shaka A.J. (1997). Application of phase-modulated CLEAN chemical EXchange spectroscopy (CLEANEX-PM) to detect water-protein proton exchange and intermolecular NOEs. J. Am. Chem. Soc..

[bib45] Hwang T.-L., van Zijl P.C.M., Mori S. (1998). Accurate quantitation of water-amide proton exchange rates using the Phase-Modulated CLEAN chemical EXchange (CLEANEX-PM) approach with a Fast-HSQC (FHSQC) detection scheme. J. Biomol. NMR.

[bib46] Jahnke W. (2002). Spin labels as a tool to identify and characterize protein - ligand interactions by NMR spectroscopy. ChemBioChem.

[bib47] Jahnke W. (2007). Perspectives of biomolecular NMR in drug discovery: the blessing and curse of versatility. J. Biomol. NMR.

[bib48] Jahnke W., Rüdisser S., Zurini M. (2001). Spin label enhanced NMR screening. J. Am. Chem. Soc..

[bib49] Jencks W.P. (1982). On the attribution and additivity of binding energies. Proc. Natl. Acad. Sci. U. S. A..

[bib50] Jubb H., Higueruelo A.P., Winter A., Blundell T.L. (2012). Structural biology and drug discovery for protein–protein interactions. Trends Pharmacol. Sci..

[bib51] Karplus M., Kuriyan J. (2005). Molecular dynamics and protein function. Proc. Natl. Acad. Sci. U. S. A..

[bib52] Kay L.E. (1998). Protein dynamics from NMR. Nat. Struct. Biol..

[bib53] Kay L.E., Torchia D.A., Bax A. (1989). Backbone dynamics of proteins as studied by nitrogen-15 inverse detected heteronuclear NMR spectroscopy: application to staphylococcal nuclease. Biochemistry.

[bib54] Kern D., Zuiderweg E. (2003). The role of dynamics in allosteric regulation. Curr. Opin. Struct. Biol..

[bib55] Krzeminski M., Loth K., Boelens R., Bonvin A.M.J.J. (2010). SAMPLEX: automatic mapping of perturbed and unperturbed regions of proteins and complexes. BMC Bioinforma..

[bib56] Kumari S., van der Hoorn R.A. (2011). A structural biology perspective on bioactive small molecules and their plant targets. Curr. Opin. Plant Biol..

[bib57] Kuntz I.D., Chen K., Sharp K.A., Kollman P.A. (1999). The maximal affinity of ligands. Proc. Natl. Acad. Sci. U. S. A.

[bib58] Lakomek N.-A., Ying J., Bax A. (2012). Measurement of ^15^N relaxation rates in perdeuterated proteins by TROSY-based methods. J. Biomol. NMR.

[bib59] Lepre C.A. (2011). Practical aspects of NMR-based fragment screening. Methods Enzymol..

[bib60] Li Y., Altorelli N.L., Bahna F., Honig B., Shapiro L., Palmer A.G. (2013). Mechanism of E-cadherin dimerization probed by NMR relaxation dispersion. Proc. Natl. Acad. Sci. U. S. A.

[bib61] Lin D., Sze K.H., Cui Y., Zhu G. (2002). Clean SEA-HSQC: a method to map solvent exposed amides in large non-deuterated proteins with gradient-enhanced HSQC. J. Biomol. NMR.

[bib62] Lipari G., Szabo A. (1982). Model-free approach to the interpretation of nuclear magnetic resonance relaxation in macromolecules. 1. Theory and range of validity. J. Am. Chem. Soc..

[bib63] Long D., Marshall C.B., Bouvignies G., Mazhab-Jafari M.T., Smith M.J., Ikura M., Kay L.E. (2013). A comparative CEST NMR study of slow conformational dynamics of small GTPases complexed with GTP and GTP analogues. Angew. Chem. Int. Ed..

[bib64] Ludwig C., Guenther U.L. (2009). Ligand based NMR methods for drug discovery. Front. Biosci..

[bib65] Mainz A., Religa T.L., Sprangers R., Linser R., Kay L.E., Reif B. (2013). NMR spectroscopy of soluble protein complexes at one mega-Dalton and beyond. Angew. Chem. Int. Ed..

[bib66] Makley L.N., Gestwicki J.E. (2013). Expanding the number of “druggable” targets: non-enzymes and protein-protein interactions. Chem. Biol. Drug. Des..

[bib67] Mashalidis E.H., Sledź P., Lang S., Abell C. (2013). A three-stage biophysical screening cascade for fragment-based drug discovery. Nat. Protoc..

[bib68] Mayer M., Meyer B. (1999). Characterization of ligand binding by saturation transfer difference NMR spectroscopy. Angew. Chem. Int. Ed..

[bib69] Mayer M., Meyer B. (2001). Group epitope mapping by saturation transfer difference NMR to identify segments of a ligand in direct contact with a protein receptor. J. Am. Chem. Soc..

[bib70] Mittermaier A., Kay L.E. (2006). New tools provide new insights in NMR studies of protein dynamics. Science.

[bib71] Mootz H.D. (2009). Split inteins as versatile tools for protein semisynthesis. ChemBioChem.

[bib72] Mortenson P.N., Murray C.W. (2011). Assessing the lipophilicity of fragments and early hits. J. Comput Aided Mol. Des..

[bib73] Mulder F.A.A., Skrynnikov N.R., Hon B., Dahlquist F.W., Kay L.E. (2001). Measurement of slow (μs−ms) time scale dynamics in protein side chains by ^15^N relaxation dispersion NMR spectroscopy: application to Asn and Gln residues in a cavity mutant of T4 lysozyme. J. Am. Chem. Soc..

[bib74] Mund M., Overbeck J.H., Ullmann J., Sprangers R. (2013). LEGO-NMR spectroscopy: a method to visualize individual subunits in large heteromeric complexes. Angew. Chem. Int. Ed..

[bib75] Muona M., Aranko A.S., Raulinaitis V., Iwaï H. (2010). Segmental isotopic labeling of multi-domain and fusion proteins by protein trans-splicing in vivo and in vitro. Nat. Protoc..

[bib76] Murray C.W., Verdonk M.L. (2002). The consequences of translational and rotational entropy lost by small molecules on binding to proteins. J. Comput Aided Mol. Des..

[bib77] Nalepa G., Rolfe M., Harper J.W. (2006). Drug discovery in the ubiquitin–proteasome system. Nat. Rev. Drug. Discov..

[bib78] Neudecker P., Lundström P., Kay L.E. (2009). Relaxation dispersion NMR spectroscopy as a tool for detailed studies of protein folding. Biophys. J..

[bib79] Nooren I.M.A., Thornton J.M. (2003). Diversity of protein-protein interactions. EMBO J..

[bib80] Núñez S., Venhorst J., Kruse C.G. (2012). Target–drug interactions: first principles and their application to drug discovery. Drug. Discov. Today.

[bib81] Ollerenshaw J.E., Tugarinov V. (2003). Methyl TROSY: explanation and experimental verification. Magn. Reson. Chem..

[bib82] Oost T.K., Sun C., Armstrong R.C., Al-Assaad A.-S., Betz S.F., Deckwerth T.L., Ding H., Elmore S.W., Meadows R.P., Olejniczak E.T., Oleksijew A., Oltersdorf T., Rosenberg S.H., Shoemaker A.R., Tomaselli K.J., Zou H., Fesik S.W. (2004). Discovery of potent antagonists of the antiapoptotic protein XIAP for the treatment of cancer. J. Med. Chem..

[bib83] Palmer A.G. (2004). NMR characterization of the dynamics of biomacromolecules. Chem. Rev..

[bib84] Pellecchia M., Meininger D., Shen A.L., Jack R., Kasper C.B., Sem D.S. (2001). SEA-TROSY (solvent exposed amides with TROSY): a method to resolve the problem of spectral overlap in very large proteins. J. Am. Chem. Soc..

[bib85] Pellecchia M., Sem D.S., Wüthrich K. (2002). NMR in drug discovery. Nat. Rev. Drug. Discov..

[bib86] Pellecchia M., Bertini I., Cowburn D., Dalvit C., Giralt E., Jahnke W., James T.L., Homans S.W., Kessler H., Luchinat C., Meyer B., Oschkinat H., Peng J., Schwalbe H., Siegal G. (2008). Perspectives on NMR in drug discovery: a technique comes of age. Nat. Rev. Drug. Discov..

[bib87] Pervushin K., Riek R., Wider G., Wüthrich K. (1997). Attenuated T2 relaxation by mutual cancellation of dipole-dipole coupling and chemical shift anisotropy indicates an avenue to NMR structures of very large biological macromolecules in solution. Proc. Natl. Acad. Sci. U. S. A.

[bib88] Petros A.M., Huth J.R., Oost T., Park C.-M., Ding H., Wang X., Zhang H., Nimmer P., Mendoza R., Sun C., Mack J., Walter K., Dorwin S., Gramling E., Ladror U., Rosenberg S.H., Elmore S.W., Fesik S.W., Hajduk P.J. (2010). Discovery of a potent and selective Bcl-2 inhibitor using SAR by NMR. Bioorg. Med. Chem. Lett..

[bib89] Rees D.C., Congreve M., Murray C.W., Carr R. (2004). Fragment-based lead discovery. Nat. Rev. Drug. Discov..

[bib90] Roberts G.C.K. (2000). Applications of NMR in drug discovery. Drug. Discov. Today.

[bib91] Sanchez-Pedregal V.M., Reese M., Meiler J., Blommers M.J., Griesinger C., Carlomagno T. (2005). The INPHARMA method: protein-mediated interligand NOEs for pharmacophore mapping. Angew. Chem. Int. Ed..

[bib92] Sattler M., Fesik S.W. (1996). Use of deuterium labeling in NMR: overcoming a sizeable problem. Structure.

[bib93] Showalter S.A., Brüschweiler R. (2007). Validation of molecular dynamics simulations of biomolecules using NMR spin relaxation as benchmarks: application to the AMBER99SB force field. J. Chem. Theory Comput..

[bib94] Shuker S.B., Hajduk P.J., Meadows R.P., Fesik S.W. (1996). Discovering high-affinity ligands for proteins: SAR by NMR. Science.

[bib95] Sledz P., Silvestre H.L., Hung A.W., Ciulli A., Blundell T.L., Abell C. (2010). Optimization of the interligand overhauser effect for fragment linking: application to inhibitor discovery against *Mycobacterium tuberculosis* pantothenate synthetase. J. Am. Chem. Soc..

[bib96] Sledź P., Abell C., Ciulli A. (2012). NMR of Biomolecules: Towards Mechanistic Systems Biology.

[bib97] Smith M.C., Gestwicki J.E. (2012). Features of protein–protein interactions that translate into potent inhibitors: topology, surface area and affinity. Expert Rev. Mol. Med..

[bib98] Stone M.J. (2001). NMR relaxation studies of the role of conformational entropy in protein stability and ligand binding. Acc. Chem. Res..

[bib99] Stukalov A., Superti-Furga G., Colinge J. (2012). Deconvolution of targeted protein–protein interaction maps. J. Proteome Res..

[bib100] Sun Q., Burke J.P., Phan J., Burns M.C., Olejniczak E.T., Waterson A.G., Lee T., Rossanese O.W., Fesik S.W. (2012). Discovery of small molecules that bind to K-Ras and inhibit Sos-mediated activation. Angew. Chem. Int. Ed..

[bib101] Surade S., Blundell T.L. (2012). Structural biology and drug discovery of difficult targets: the limits of ligandability. Chem. Biol..

[bib102] Takahashi H., Nakanishi T., Kami K., Arata Y., Shimada I. (2000). A novel NMR method for determining the interfaces of large protein-protein complexes. Nat. Struct. Biol..

[bib103] Thangudu R.R., Bryant S.H., Panchenko A.R., Madej T. (2012). Modulating protein–protein interactions with small molecules: the importance of binding hotspots. J. Mol. Biol..

[bib104] Thiel P., Kaiser M., Ottmann C. (2012). Small-molecule stabilization of protein-protein interactions: an underestimated concept in drug discovery?. Angew. Chem. Int. Ed..

[bib105] Tugarinov V., Kay L.E. (2004). An isotope labeling strategy for methyl TROSY spectroscopy. J. Biomol. NMR.

[bib106] Tugarinov V., Sprangers R., Kay L.E. (2004). Line narrowing in methyl-TROSY using zero-quantum ^1^H-13C NMR spectroscopy. J. Am. Chem. Soc..

[bib107] Tugarinov V., Kay L.E., Ibraghimov I., Orekhov V.Y. (2005). High-resolution four-dimensional ^1^H-13C NOE spectroscopy using methyl-TROSY, sparse data acquisition, and multidimensional decomposition. J. Am. Chem. Soc..

[bib108] Tzeng S.-R., Kalodimos C.G. (2012). Protein activity regulation by conformational entropy. Nature.

[bib109] Vallurupalli P., Bouvignies G., Kay L.E. (2011). Increasing the exchange time-scale that can be probed by CPMG relaxation dispersion NMR. J. Phys. Chem. B.

[bib110] Van Molle I., Thomann A., Buckley D.L., So E.C., Lang S., Crews C.M., Ciulli A. (2012). Dissecting fragment-based lead discovery at the von Hippel-Lindau protein:Hypoxia inducible factor 1α protein-protein interface. Chem. Biol..

[bib111] Vanwetswinkel S., Heetebrij R.J., van Duynhoven J., Hollander J.G., Filippov D.V., Hajduk P.J., Siegal G. (2005). TINS, target immobilized NMR screening: an efficient and sensitive method for ligand discovery. Chem. Biol..

[bib112] Velyvis A., Schachman H.K., Kay L.E. (2009). Application of methyl-TROSY NMR to test allosteric models describing effects of nucleotide binding to aspartate transcarbamoylase. J. Mol. Biol..

[bib113] Wang C., Grey J., M., G, Palmer A. (2001). CPMG sequences with enhanced sensitivity to chemical exchange. J. Biomol. NMR.

[bib114] Wells J.A., McClendon C.L. (2007). Reaching for high-hanging fruit in drug discovery at protein–protein interfaces. Nature.

[bib115] Williamson M.P. (2013). Using chemical shift perturbation to characterise ligand binding. Prog. Nucl. Magn. Reson. Spectrosc..

[bib116] Woodhead A.J., Angove H., Carr M.G., Chessari G., Congreve M., Coyle J.E., Cosme J., Graham B., Day P.J., Downham R., Fazal L., Feltell R., Figueroa E., Frederickson M., Lewis J., McMenamin R., Murray C.W., O'Brien M.A., Parra L., Patel S., Phillips T., Rees D.C., Rich S., Smith D.-M., Trewartha G., Vinkovic M., Williams B., Woolford A.J. (2010). Discovery of (2,4-Dihydroxy-5-isopropylphenyl)-[5-(4-methylpiperazin-1-ylmethyl)-1,3-dihydroisoindol-2-yl]methanone (AT13387), a novel inhibitor of the molecular chaperone Hsp90 by fragment based drug design. J. Med. Chem..

[bib117] Zhu Z., Sun Z.-Y., Ye Y., Voigt J., Strickland C., Smith E.M., Cumming J., Wang L., Wong J., Wang Y.-S., Wyss D.F., Chen X., Kuvelkar R., Kennedy M.E., Favreau L., Parker E., McKittrick B.A., Stamford A., Czarniecki M., Greenlee W., Hunter J.C. (2010). Discovery of cyclic acylguanidines as highly potent and selective beta-site amyloid cleaving enzyme (BACE) inhibitors: Part I–inhibitor design and validation. J. Med. Chem..

[bib118] Zorn J.A., Wells J.A. (2010). Turning enzymes on with small molecules. Nat. Chem. Biol..

